# Socio‐economic distribution of e‐cigarette use among recent former regular smokers and current smokers at ages 25–26 in England

**DOI:** 10.1111/add.15345

**Published:** 2021-01-04

**Authors:** Thierry Gagné, Jamie Brown

**Affiliations:** ^1^ Department of Epidemiology and Public Health University College London London UK; ^2^ Department of Behavioural Science and Health, Co‐Director, Tobacco and Alcohol Research Group University College London London UK; ^3^ SPECTRUM Consortium London UK

**Keywords:** E‐cigarettes, England, Next Steps study, Smoking, Socioeconomic Factors, young adults

## Abstract

**Background and aims:**

E‐cigarettes may potentially help young adult smokers to quit smoking, yet little is known about differences among socio‐economic groups. We examined associations between key socio‐economic characteristics and e‐cigarette use among recent former smokers and current smokers in a sample of young adults in England.

**Design, setting, participants and measurements:**

We used data on 346 recent former regular (daily for 12+ months) smokers and 1913 current smokers from the ages 25–26 wave of the Next Steps cohort study (2015–2016). In multinomial logistic regression, we estimated relative risk ratios (RRR) of e‐cigarette use (never, former, non‐daily, daily) by educational attainment, social class [using the National Statistics Socio‐economic classification (NS‐SEC)] and employment status [full‐time, part‐time, unemployed and other ‘inactivity’ (e.g. stay‐at‐home parents and permanantly disabled)], adjusting for sex.

**Findings:**

Among recent former regular smokers, there were no patterns of association between socio‐economic characteristics and e‐cigarette use. Among current smokers: (1) compared with higher occupation (NS‐SEC I/II), intermediate occupation (NS‐SEC III/IV) was positively associated with non‐daily e‐cigarette use [RRR = 1.77, 95% confidence interval (CI) = 1.03–3.03]; (2) compared with full‐time employment, unemployment was negatively associated with non‐daily and daily e‐cigarette use (RRR = 0.38, 95% CI = 0.18–0.81; RRR = 0.12, 95% CI = 0.02–0.56) and other economic inactivity was negatively associated with daily e‐cigarette use (RRR = 0.39, 95% CI = 0.16–0.93).

**Conclusions:**

Among young adult smokers in England, lower‐status occupational groups were more likely to use e‐cigarettes on a non‐daily basis than to have never used compared with higher status occupational groups. Compared with people in full‐time employment, those without employment were less likely to use e‐cigarettes daily than to have never used.

## Introduction

Young adults report a higher prevalence of cigarette smoking compared with other age groups and benefit substantially from quitting smoking at this life stage [1, 2]. In the United Kingdom, young adults ages 25–34 years are 31% more likely to smoke cigarettes relative to the adult population [2]. Young adults who attempt to quit are also less likely to quit successfully compared to older age groups, as quit success among past‐year smokers attempting to stop in England have been on average 19% lower among young adults aged 18–34 compared with adults aged 55+ over the past 15 years [3]. Despite this, evidence on effective smoking cessation interventions in this age group is limited [4, 5, 6, 7].

The use of electronic nicotine delivery devices (ENDS), namely e‐cigarettes, has the potential to help smokers to reduce and quit smoking [8, 9, 10]. Studies suggest that adults who use e‐cigarettes on a daily basis report higher rates of smoking cessation and lower smoking frequency [11, 12, 13, 14, 15]. Non‐daily e‐cigarette use, however, continues to represent an important risk factor for continued smoking [12, 13, 14]. In young adults, a single trial with 99 daily smokers found that using nicotine e‐cigarettes (compared with placebo e‐cigarettes) helped them to reduce by half their cigarette consumption [16]. Observational studies have suggested that young adults may be less likely to use e‐cigarettes to quit smoking because they: (1) were the least likely age group to seek nicotine replacement therapies and (2) did not seek e‐cigarettes as a cessation aid [17]. Use of e‐cigarettes, however, has been associated with higher abstinence rates following a quit attempt in England in both younger (16–44) and older adults (45 and over) [18].

An important extension of this debate concerns the capacity of e‐cigarettes to be used for smoking cessation by socially disadvantaged smokers and contribute to the reduction of social inequalities in smoking and smoking‐attributable diseases [19]. Smoking is disproportionally distributed among those who lack qualifications, are unemployed or employed in routine occupations, including among young adults [20, 21, 22]. Trends over time also suggest that these social inequalities in smoking are not decreasing in the United Kingdom [2]. Evidence on the socio‐economic distribution of e‐cigarette use, however, is slim.

Hartwell *et al*. performed the first review on this issue and found that whereas higher education was associated with the initiation of e‐cigarettes, there were no associations between socio‐economic characteristics and current use [23]. Lucherini *et al*. updated this review, and argued that there was evidence from the United States that current e‐cigarette use increased in lower socio‐economic groups and was reaching the level of use found first among more privileged early adopters [24, 25, 26]. They noted, however, that this trend was not systematically replicated in other countries with different products, regulations and smoking populations. They concluded their review by highlighting the continued ‘lack of direct evidence on the likely impact of e‐cigarettes on inequalities in smoking’ ([24], p. 9).

Two overarching issues also limit the applicability of the evidence. First, the majority of studies included in reviews used data collected more than 6–7 years ago. Substantial changes have occurred in the nature of e‐cigarette devices and the characteristics of e‐cigarette users over time, which are moving from early adopters to the rest of the smoking population [24, 27]. Secondly, the majority of these studies used simplistic definitions of frequency such as ‘current’ use, thereby obfuscating differences between regular and irregular use, and did not distinguish the distribution of e‐cigarette use among smokers and non‐smokers [11, 28]. For instance, studies in the United Kingdom found that smokers were more likely to use e‐cigarettes if they were employed in a higher occupational class [29, 30]. Without distinguishing its frequency, we cannot parse out the implications of findings on the reduction of social inequalities in smoking. To the best of our knowledge, no studies have as yet explored this in young adults.

### Objective

To assess the socio‐economic distribution of e‐cigarette use in the young adult population, we examined the association between key socio‐economic characteristics—educational attainment, social class and employment status—and e‐cigarette use (never, former, non‐daily and daily) among recent former regular smokers and current smokers in a national sample of young adults ages 25–26 in England (2015–16).

## Methods

### Data

We used data from the latest wave of the Next Steps cohort study, formerly known as the Longitudinal Study of Young People in England (LSYPE) [31, 32]. This study recruited 15 770 young people born between September 1989 and August 1990 aged 13–14 in 2004 and invited them to participate each subsequent year until ages 19–20, and another time at ages 25–26 between August 2015 and September 2016, for a total of eight waves. The baseline sample design was stratified by schools, oversampled deprived schools, and ensured that within a deprivation stratum, all pupils within an ethnic group had an equal chance of selection. The study was initially commissioned by the UK Department of Education to study the successful progression from compulsory education. With the ages 25–26 wave, the UCL Centre for Longitudinal Studies (CLS) took up management and extended the focus of the cohort to a wider range of outcomes, including health. A total of 7707 cohort members participated in the latest wave, representing a 51% response rate compared with baseline. We note that there was attrition by smoking status, as baseline participants who smoked were 28% less likely to have participated at ages 25–26. To mitigate potential bias from attrition, the Next Steps team provides a longitudinal weight based on variables associated with attrition across the study: parents’ education, employment, occupation and marital status at ages 13–14, participants’ sex, ethnicity, region and special educational needs at ages 13–14 and participants’ housing tenure, economic activity and cannabis use history at ages 19–20 [31].

### Measures

We measured e‐cigarette use and smoking status at ages 25–26 using two single items, one concerning cigarettes and the other e‐cigarettes: ‘Which of the following statements applies to you? (1) I have never tried e‐cigarettes/cigarettes; (2) I have tried e‐cigarettes/cigarettes but don't use them at all now; (3) I now use e‐cigarettes/cigarettes occasionally but not every day; and (4) I use e‐cigarettes/cigarettes every day’. The proportion of missing data on these items was 3.7% each. We categorized participants to be: (1) never, (2) former, (3) non‐daily and (4) daily e‐cigarette users and cigarette smokers. We measured cessation behaviour using two other items: ‘Have you ever smoked cigarettes regularly—regularly meaning at least one cigarette a day for 12 months or more?’ and ‘How old were you when you last smoked cigarettes regularly?’. The proportion of missing data on the two items among smokers was 0.1% each. We defined former regular smokers as ‘recent’ if they last smoked regularly (daily for 12+ months) within the past 2 years when subtracting from their age the age at which they stopped smoking regularly (e.g. age 23+ if they were age 25, age 24+ if they were age 26). We therefore examined the socio‐economic distribution of e‐cigarette use in two groups: (1) ‘recent former regular smokers’, who stopped smoking regularly within the past 2 years and did not currently smoke (*n* = 346); and (2) ‘current smokers’, including non‐daily and daily smokers (*n* = 1913).

We measured educational attainment based on the participants’ National Vocational Qualification (NVQ) equivalent: (1) no qualifications, (2) NVQ 1–2: secondary education (age 16), (3) NVQ 3: A‐levels (pre‐university, ages 17–18), (4) NVQ 4–5: further or higher education (university, ages 19+). We measured social class using the National Statistics Socio‐economic Classification (NS‐SEC), a classification constructed to measure the employment relations and conditions of occupations that we reduced from seven to three main categories: (1) NS‐SEC 1‐2: higher; (2) NS‐SEC 3‐4: intermediate; (3) NS‐SEC 5–7: lower; and (4) a residual category for the non‐employed [33, 34]. We finally measured employment status using a variable derived by the Next Steps team and recoded it as: (1) full‐time, (2) part‐time, (3) unemployed and (4) ‘other’ inactivity, including, e.g. stay‐at‐home parents, those in full‐time studies or in permanent disability. The proportion of missing data on these variables varied from 0.0 to 0.7%.

Building upon the literature regarding correlates of e‐cigarettes, we considered the following control variables: (1) year (2015/2016); (2) sex (male/female); (3) ethnic group (white/non‐white) and at the study baseline (ages 13–14); (4) urbanization (≥ 10 000 inhabitants/not); 5) disability (yes/no); (6) the main parent's age when last left education (ages ≤ 16, ages ≥ 17); (7) family structure (living with both parents/not); and (8) parents’ home ownership (owning/not) [ 23, 24]. We also considered adolescent smoking (yes/no), categorizing participants to have been smokers (smoker/non‐smoker) if they answered ‘I sometimes smoke cigarettes now but I don't smoke as many as one a week’, ‘I usually smoke between one and six cigarettes a week’ or ‘I usually smoke more than six cigarettes a week’ in at least one of the first three waves (ages 13–14, 14–15 and 15–16).

### Statistical analyses

We first described the distribution of e‐cigarette use and cigarette smoking in the sample. We then regressed e‐cigarette use separately among recent former regular smokers and current smokers using sequential multinomial logistic models, entering: (model 1) education, (model 2) education + social class and (model 3) education + employment status. We used sequential models to distinguish the ‘total effect’ of education and its ‘direct effect’ not explained through social class or employment status on e‐cigarette use [35]. We did not include a model with social class and employment status together, as they are co‐linear, i.e. those not in employment have the same social class value: ‘not applicable’. We report relative risk ratios (RRR) across categories of independent variables, their 95% confidence interval (CI) and the joint test of association across outcomes categories for each independent variable (using the Stata *test* command).

To limit the number of covariates and prevent overfitting, we preliminarily examined associations between the selected control variables and e‐cigarette use with the intention to keep variables associated with e‐cigarette use at the *P* < 0.05 level, including with their joint test of association. We therefore only added sex in models among recent former regular smokers and current smokers (Supporting information, Table S1). We adjusted all analyses for the survey design using the cluster and stratification variables and for non‐response and attrition using the longitudinal weight variable created by the Next Steps team. Analyses were produced using listwise deletion in Stata version14 [36]. The proportion of dropped cases in complete‐case analyses was at maximum 0.0% among recent former regular smokers and 0.4% among current smokers.

## Results

### Sample characteristics

Table 1 reports the distribution of e‐cigarette use among recent former regular smokers and current smokers across the categories of educational attainment, social class and employment status at ages 25–26 in 2015–16. In the full sample of 7707 participants, 4281 (55.6%) were female and 5255 (69.5%) identified as white. For education, 534 (7.1%) did not obtain any qualifications and 2414 (31.4%) only completed secondary education. Similarly, 1701 (22.1%) were employed in a lower occupation and 502 (6.6%) were unemployed. Supporting the magnitude of social inequalities in smoking in this group, young adults were 133% more likely to smoke if they had no qualifications compared to those with further or higher education (46.2 versus 19.8%), 55% more likely to smoke if they were employed in a lower occupation compared to those in higher occupations (38.1 versus 24.6%) and 59% more likely to smoke if they were unemployed compared to those in full‐time employment (46.0 versus 29.0%) (Supporting information, Table S2).

**Table 1:** Description of e‐cigarette use among recent former regular smokers and current smokers (ages 25–26); Next Steps study, 2015–16

E‐cigarette use	Recent former regular smokers *n* = 346								Current smokers *n* = 1913							
	Never		Former		Non‐daily		Daily		Never		Former		Non‐daily		Daily	
Variables	*n*	%	*n*	%	*n*	%	*n*	%	*n*	%	*n*	%	*n*	%	*n*	%
Whole sample	110	31.8	129	37.3	25	7.2	82	23.7	658	34.4	929	48.6	225	11.8	101	5.3
Education (NVQ)																
No qualifications	10	33.3	9	30.0	5	16.7	6	20.0	71	36.4	91	46.7	22	11.3	11	5.6
1–2: Secondary	40	27.0	54	36.5	9	6.1	45	30.4	282	34.0	388	46.8	116	14.0	43	5.2
3: A‐levels	14	29.2	19	39.6	4	8.3	11	22.9	108	32.8	172	52.3	29	8.8	20	6.1 4.9
4–5: FE/HE	46	38.3	47	39.2	7	5.8	20	16.7	193	35.0	275	49.8	57	10.3	27	
Missing	0		0		0		0		4		3		1		0	
Social class (NS‐SEC)																
1–2: Higher	35	31.0	45	39.8	5	4.4	28	24.8	207	33.6	310	50.3	57	9.3	42	6.8
3–4: Intermediate	26	37.7	23	33.3	5	7.2	15	21.7	105	32.5	155	48.0	49	15.2	14	4.3
5–7: Lower	20	20.8	40	41.7	7	7.3	29	30.2	174	31.0	276	49.2	78	13.9	33	5.9
Not applicable	29	42.6	21	30.9	8	11.8	10	14.7	172	41.6	188	45.5	41	9.9	12	2.9
Missing	0		0		0		0		0		0		0		0	
Employment status																
FT employed	67	27.6	93	38.3	16	6.6	67	27.6	396	31.5	623	49.6	156	12.4	81	6.4
PT employed	13	38.2	14	41.2	1	2.9	6	17.6	86	36.4	113	47.9	28	11.9	9	3.8
Unemployed	8	36.4	7	31.8	4	18.2	3	13.6	71	42.8	81	48.8	12	7.2	2	1.2
Other	22	46.8	15	31.9	4	8.5	6	12.8	104	41.4	110	43.8	28	11.2	9	3.6
Missing	0		0		0		0		1		2		1		0	

NVQ = National Vocational Qualification; FE/HE = further or higher education; NS‐SEC = National Statistics Socio‐economic Classification; FT = full‐time; PT = part‐time. The % column represents the prevalence of e‐cigarette use categories across the categories of socio‐economic characteristics. Participants with a ‘Not applicable’ social class were not employed.

Regarding smoking, 4306 (58.0%) had never tried cigarettes, 1202 (15.6%) were former smokers, 818 (10.6%) were non‐daily smokers and 1095 (14.2%) were daily smokers. Regarding e‐cigarette use, 5427 (73.1%) had never tried e‐cigarettes, 1483 (19.2%) were former e‐cigarette users, 224 (2.9%) were non‐daily users and 291 (3.8%) were daily users. Comparing e‐cigarette use across smoking status categories, non‐daily e‐cigarette use was more prevalent among daily smokers (13.0%) and non‐daily smokers (11.2%) compared to former smokers (4.6%). Daily e‐cigarette use was less prevalent among daily smokers (2.8%) compared to non‐daily smokers (9.1%) and former smokers (11.6%). Only 19 (0.3% of the full sample) participants used e‐cigarettes while having never tried cigarettes (Supporting information, Table S3).

Among the analytical group of recent former regular smokers, 129 (37.3%), 25 (7.2%) and 82 (23.7%) were former, non‐daily and daily e‐cigarette users, respectively. Among the analytical group of current smokers, 929 (48.6%), 225 (11.7%) and 101 (5.3%) participants were former, non‐daily and daily e‐cigarette users, respectively. Regarding smoking frequency, 57.2% of current smokers were daily smokers.

### Distribution of e‐cigarette use among recent former regular smokers and current smokers

Table 2 presents the sex‐adjusted weighted relative risk ratios (RRR) of using e‐cigarettes formerly, non‐daily or daily among recent former regular smokers at ages 25–26 according to educational attainment, social class and employment status. We found that neither educational attainment (*P* = 0.437), social class (*P* = 0.733) and employment status (*P* = 0.724) were associated with e‐cigarette use in this group. Looking at exposure–outcome combinations, in model 1 with education, recent former regular smokers who only completed secondary education had a 125% higher relative risk of using e‐cigarettes daily compared to those with a further or higher education degree (RRR = 2.25, 95% CI = 1.00–5.04, *P* = 0.050).

**Table 2:** Socio‐economic distribution of e‐cigarette use among recent former regular smokers at ages 25–26 in England; Next Steps study, 2015–16

	Model 1, *n* = 346						Model 2, *n* = 346						Model 3, *n* = 346					
E‐cigarette use	Former		Non‐daily		Daily		Former		Non‐daily		Daily		Former		Non‐daily		Daily	
	RRR	95% CI	RRR	95% CI	RRR	95% CI	RRR	95% CI	RRR	95% CI	RRR	95% CI	RRR	95% CI	RRR	95% CI	RRR	95% CI
Education (NVQ)	Joint test *P =* 0.437						Joint test *P =* 0.585						Joint test *P =* 0.539					
No qualifications	0.96	(0.33–2.81)	2.10	(0.49–9.10)	1.61	(0.42–6.14)	1.07	(0.37–3.12)	1.83	(0.40–8.37)	1.80	(0.44–7.37)	1.08	(0.36–3.30)	2.06	(0.44–9.75)	1.87	(0.49–7.11)
1–2: Secondary	1.44	(0.74–2.81)	0.76	(0.24–2.45)	2.25	(1.00–5.04)	1.30	(0.65–2.60)	0.65	(0.19–2.17)	2.07	(0.88–4.88)	1.46	(0.74–2.88)	0.84	(0.26–2.73)	2.25	(0.98–5.14)
3: A‐levels	1.14	(0.50–2.59)	1.03	(0.24–4.34)	1.53	(0.58–4.04)	1.10	(0.47–2.55)	0.99	(0.24–4.22)	1.50	(0.56–4.01)	1.14	(0.50–2.61)	1.15	(0.26–5.10)	1.52	(0.56–4.10)
4–5: FE/HE (ref.)	–		–		–		–		–		–		–		–		–	
Social class (NS‐SEC)							Joint test *P =* 0.733											
1–2: Higher (ref.)							–		–		–							
3–4: Intermediate							0.85	(0.39–1.86)	0.95	(0.26–3.96)	1.01	(0.36–2.87)						
5–7: Lower							1.38	(0.62–3.09)	1.96	(0.50–7.70)	1.29	(0.48–3.49)						
Not applicable							0.69	(0.30–1.58)	1.82	(0.50–6.59)	0.61	(0.21–1.75)						
Employment status													Joint test *P =* 0.724					
FT employed (ref.)													–		–		–	
PT employed													0.92	(0.38–2.26)	0.58	(0.06–5.19)	0.91	(0.29–2.85)
Unemployed													0.76	(0.20–2.81)	2.3	(0.56–9.46)	0.61	(0.13–2.86)
Other													0.62	(0.28–1.34)	1.03	(0.27–3.99)	0.48	(0.17–1.30)

Estimates are relative risk ratios (RRR) from multinomial logistic models adjusted for complex sampling and non‐response, controlling for sex. The outcome reference category is having never initiated e‐cigarettes. Model 1 enters education separately, model 2 enters education + social class and model 3 enters education + employment status. ‘Joint test *P*’ refers to the joint test of significance of coefficients across the outcome categories. Participants with a ‘Not applicable’ social class were not employed. NVQ = National Vocational Qualification; FE/HE = further or higher education; NS‐SEC = National Statistics Socio‐economic Classification; FT = full‐time; PT = part‐time; CI= confidence interval.

Table 3 presents the sex‐adjusted weighted relative risk ratios of using e‐cigarettes formerly, non‐daily or daily among current smokers at ages 25–26 according to educational attainment, social class and employment status. We found that two of the three socio‐economic characteristics—social class (*P* = 0.024) and employment status (*P* = 0.041)—were associated with the risk of using e‐cigarettes in this group, whereas educational attainment was not associated with e‐cigarette use (*P* = 0.501). For non‐daily e‐cigarette use, we found in model 2 with social class that, compared with those in a higher occupational class, those in an intermediate occupational class had a 77% higher relative risk of using e‐cigarettes non‐daily (RRR = 1.77, 95% CI = 1.03–3.03, *P* = 0.037) and those in a lower occupational class had a 62% higher relative risk of using e‐cigarettes non‐daily (RRR = 1.62, 95% CI = 1.00–2.61, *P* = 0.050). We also found in model 3 with employment status that, compared to those employed full‐time, those unemployed had a 62% lower relative risk of using e‐cigarettes non‐daily (RRR = 0.38, 95% CI = 0.18–0.81, *P* = 0.012). For daily e‐cigarette use, we found in model 2 with social class that, compared with those in a higher occupational class, those without an occupation had a 72% lower relative risk of using e‐cigarettes daily (RRR = 0.28, 95% CI = 0.12–0.66, *P* = 0.004). Finally, we found in model 3 with employment status that, compared to those employed full‐time, those unemployed had a 88% lower relative risk of using e‐cigarettes non‐daily (RRR = 0.12, 95% CI = 0.02–0.56, *P* = 0.007) and those economically inactive had a 61% lower relative risk of using e‐cigarettes non‐daily (RRR = 0.39, 95% CI = 0.16–0.93, *P* = 0.033).

**Table 3:** Socio‐economic distribution of e‐cigarette use among current smokers at ages 25–26 in England; Next Steps study, 2015–16

E‐cigarette use	Model 1, *n* = 1905						Model 2, *n* = 1905						Model 3, *n* = 1901					
	Former		Non‐daily		Daily		Former		Non‐daily		Daily		Former		Non‐daily		Daily	
	RRR	95% CI	RRR	95% CI	RRR	95% CI	RRR	95% CI	RRR	95% CI	RRR	95% CI	RRR	95% CI	RRR	95% CI	RRR	95% CI
Education (NVQ)	Joint test *P =* 0.501						Joint test *P =* 0.584						Joint test *P =* 0.337					
No qualifications	0.83	(0.54–1.26)	1.08	(0.56–2.07)	0.98	(0.39–2.44)	0.89	(0.57–1.38)	1.12	(0.58–2.16)	1.37	(0.50–3.75)	0.92	(0.60–1.41)	1.25	(0.64–2.43)	1.41	(0.52–2.44)
1–2: Secondary	0.97	(0.73–1.27)	1.21	(0.80–1.81)	0.91	(0.50–1.66)	1.00	(0.74–1.35)	1.15	(0.76–1.74)	1.12	(0.57–2.19)	1.01	(0.76–1.34)	1.33	(0.88–2.01)	1.10	
3: A‐levels	1.09	(0.76–1.56)	0.70	(0.38–1.28)	0.77	(0.36–1.68)	1.08	(0.75–1.56)	0.67	(0.37–1.24)	0.78	(0.36–1.72)	1.10	(0.76–1.58)	0.70	(0.38–1.29)	0.77	(0.35–1.69)
4–5: FE/HE (ref.)	–		–		–		–		–		–		–		–		–	
Social class (NS‐SEC)							Joint test *P =* 0.024											
1–2: Higher (ref.)							–		–		–							
3–4: Intermediate							1.08	(0.74–1.56)	**1.77**	(**1.03–3.03**)	0.66	(0.30–1.48)						
5–7: Lower							1.05	(0.75–1.46)	1.62	(1.00–2.61)	0.81	(0.45–1.47)						
Not applicable							0.77	(0.53–1.11)	0.75	(0.44–1.27)	**0.28**	(**0.12–0.66**)						
Employment status													Joint test *P =* 0.041					
FT employed (ref.)													–		–		–	
PT employed													0.90	(0.59–1.27)	0.94	(0.52–1.69)	0.47	(0.17–1.31)
Unemployed													0.70	(0.50–1.03)	**0.38**	(**0.18–0.81)**	**0.12**	(**0.02–0.56)**
Other													0.71	(0.49–1.02)	0.66	(0.37–1.20)	**0.39**	(**0.16–0.93)**

Estimates are relative risk ratios (RRR) from multinomial logistic models adjusted for complex sampling and non‐response, controlling for sex. The outcome reference category is having never initiated e‐cigarettes. Model 1 enters education separately, model 2 enters education + social class and model 3 enters education + employment status. ‘Joint test *P*' s the joint test of significance of coefficients across the outcome categories. Participants with a ‘Not applicable’ social class were not employed. NVQ = National Vocational Qualification; FE/HE = further or higher education; NS‐SEC = National Statistics Socio‐economic Classification; FT = full‐time; PT = part‐time; CI = confidence interval.

## Discussion

Examining e‐cigarette use among young adult recent former regular smokers and current smokers across key socio‐economic characteristics, we found no patterns of association among recent former regular smokers, but found that social class and employment status were associated with e‐cigarette use among current smokers. Insofar that daily e‐cigarette use has been previously associated with smoking cessation but not non‐daily use, the findings led to a mixed portrait of the potential impact of e‐cigarette use on social inequalities in smoking at the population level. Among current smokers, we found that non‐daily e‐cigarette use was more prevalent among those employed in intermediate and lower occupations, yet less prevalent among those unemployed. We also found that daily e‐cigarette use was far less frequent among current smokers who were unemployed or economically inactive.

The findings that (1) non‐daily use was more common among those in intermediate and lower occupations and (2) daily use was less likely among those unemployed or economically inactive suggest that e‐cigarettes are unlikely to have a positive impact on social inequalities in smoking among young adults. The finding regarding social class is in line with a recent review of qualitative studies on e‐cigarette use among socio‐economically disadvantaged smokers that found that whereas they were more likely to have tried e‐cigarettes, they were also more likely to have a negative perception of its capacity to help them quit smoking [37]. The associations found with unemployment and economic inactivity recall the importance of economic barriers to the tailored use of e‐cigarettes for smoking cessation [38]. In her ethnographic work, Thirlway argued that economic costs were likely to be one of the most important barriers to the use of e‐cigarettes for smoking cessation by less privileged groups in the United Kingdom [39, 40]. Despite the fact that expenditures for e‐cigarettes are often lower than for cigarettes [41], Thirlway found that money‐saving practices led those already poor to modify their consumption practices regarding e‐cigarettes, limiting its benefits for smoking cessation [40].

The lack of patterns of association with educational attainment is in line with the findings of most studies on current e‐cigarette use in the adult population [24]. A single study in Switzerland found that young adult male smokers in 2010–13 were more likely to currently use e‐cigarettes if they had fewer qualifications [42]. Perceptions related to e‐cigarette use and its capacity to support smoking cessation may be different among young adult smokers with fewer qualifications between genders [39, 43].

Our findings have two direct implications for future studies. The first concerns adding new support for the systematic use of a refined definition of e‐cigarette use beyond current use [11, 28]. Contrasting non‐daily and daily e‐cigarette use, which have different implications for cessation behaviour, have led us to a more nuanced assessment of the potential roles of social class and employment status in e‐cigarette use among smokers. The second concerns the need for studies exploring the motivations for e‐cigarette use besides cessation among young adult smokers in lower occupations and barriers to the tailored use of e‐cigarettes for cessation among young adult smokers not in employment. We note that the equity impact of daily e‐cigarette use on smoking cessation depends upon its potential influence on the risk of later relapse. Brose *et al*. found in a small longitudinal sample of UK adults that whereas non‐daily e‐cigarette use was related with a higher risk of relapse, there was no such evidence for daily e‐cigarette use [44].

### Strengths and limitations

This study builds upon the sample size, representativeness and information collected in the Next Steps cohort to inform the association between socio‐economic characteristics and e‐cigarette use in young adults in England. We note four limitations. First, the lack of data on nicotine dependence, smoking intensity, reasons for using e‐cigarettes, nicotine content and intent to quit prevented us from drawing a stronger portrait of cessation outcomes. Secondly, the cross‐sectional nature of the analysis precludes us from inferring causal relationships from the associations reported here. In particular, while daily e‐cigarette use was more prevalent among former and non‐daily smokers compared to daily smokers, we cannot confirm that daily e‐cigarette use is causally linked to smoking reduction and cessation. Thirdly, these data were collected in 2015–16 before the implementation of new laws that could impact the role of e‐cigarette use in smoking cessation, such as the European Union Tobacco Products Directive, which enforced stricter restrictions on ingredients and nicotine content in 2016–17. Tobacco control efforts since 2015–16 may also have led to different cessation behaviours in young adults across social groups [45]. Finally, the analysis was not pre‐registered, and the results should be considered exploratory.

## Conclusions

This study draws upon the evidence on the potential of e‐cigarettes for smoking reduction and cessation and the lack of evidence on the social distribution of e‐cigarette use in young adulthood. The findings suggest that e‐cigarette use among young adults is unlikely to have a positive impact on inequalities. Unemployed and economically inactive young adult smokers were less likely to use e‐cigarettes daily, adding support to the importance of economic barriers in the use of e‐cigarettes for smoking cessation. Public health initiatives interested in promoting e‐cigarettes or other electronic nicotine delivery devices as cessation aids are likely to need to tackle the specific obstacles to the appropriate use of e‐cigarettes in particular disadvantaged smokers. As young adults represent a unique life period, studies should extend the analysis of the socio‐economic distribution of non‐daily and daily e‐cigarette use amond former and current smokers in other age groups.

## Declaration of interests

J.B. has received unrestricted research funding from Pfizer to study smoking cessation outside the submitted work. J.B. does not, and will not, take funds from e‐cigarettes manufacturers or the tobacco industry.

## Supporting information


**Table S1** Association of selected covariates with e‐cigarette use at ages 25–26 in England. Next Steps study, 2015–16.
**Table S2** Socioeconomic distribution of smoking status. Next Steps study, 2015–16.
**Table S3** Distribution of e‐cigarette use and cigarette smoking. Next Steps (*n* = 7419, 2015–16).
