# Supplementary material for: Socio‐economic distribution of e‐cigarette use among recent former regular smokers and current smokers at ages 25–26 in England
Source: Addiction. 2021 Jan 4;116(6):1548–57. doi: 10.1111/add.15345 (PMC8246545; doi:10.1111/add.15345)
Supplement: Supplementary file 1 — Table S1 Association of selected covariates with e‐cigarette use at ages 25–26 in England. Next Steps study, 2015–16. Table S2 Socioeconomic distribution of smoking status. Next Steps study, 2015–16. Table S3 Distribution of e‐cigarette use and cigarette smoking. Next Steps (n = 7419, 2015–16). [file ADD-116-1548-s001.docx]

**SUPPLEMENTARY TABLE 1**

**Association of selected covariates with e-cigarette use at ages 25-26 in England. Next Steps study, 2015-16.**

|  | **Recent former regular smokers**  **(*n* = 346)** | | | | | | **Current smokers**  **(*n* = 1,913)** | | | | | |
| --- | --- | --- | --- | --- | --- | --- | --- | --- | --- | --- | --- | --- |
| **E-cigarette use** | **Former** | | **Non-daily** | | **Daily** | | **Former** | | **Non-daily** | | **Daily** | |
|  | **RRR** | ***p*** | **RRR** | ***p*** | **RRR** | ***p*** | **RRR** | ***p*** | **RRR** | ***p*** | **RRR** | ***p*** |
|  |  |  |  |  |  |  |  |  |  |  |  |  |
| **Year** | Joint test *p =* .784 | | | | | | Joint test *p =* .626 | | | | | |
| 2015 (ref.)  2016 | 1.31 | .445 | 1.30 | .66 | 0.94 | .883 | 0.97 | .830 | 1.17 | .475 | 0.75 | .348 |
| **Sex** | **Joint test *p <* .001** | | | | | | **Joint test *p =* .007** | | | | | |
| M (ref.)  F | 0.55 | .055 | 0.54 | .188 | **0.19** | **<.001** | **0.76** | **.019** | **0.59** | **.004** | **0.58** | **.038** |
| **Ethnic group** | Joint test *p =* .259 | | | | | | Joint test *p =* .128 | | | | | |
| White (ref.)  Non-White | 0.50 | .050 | 0.67 | .508 | 0.58 | .256 | **0.69** | **.027** | 1.02 | .926 | 0.89 | .717 |
| **Urbanization** | Joint test *p =* .792 | | | | | | Joint test *p =* .241 | | | | | |
| Urban (ref.)  Less urban | 0.78 | .490 | 0.73 | .618 | 1.09 | .827 | 1.34 | .068 | 1.23 | .384 | 1.60 | .132 |
| **Longstanding limitations** | Joint test *p =* .889 | | | | | | Joint test *p =* .252 | | | | | |
| No disability (ref.)  Disability | 1.01 | .981 | 1.47 | .550 | 0.84 | .743 | 0.90 | .533 | 1.07 | .788 | 0.46 | .056 |
| **Adolescent smoking (13-16)** | **Joint test *p =* .010** | | | | | | Joint test *p =* .202 | | | | | |
| Did not smoke (ref.)  Smoked | 1.65 | .098 | 2.21 | .130 | 0.59 | .137 | 1.24 | .095 | 0.89 | .535 | 0.96 | .882 |
| **Main parent’s education** | Joint test *p =* .222 | | | | | | Joint test *p =* .830 | | | | | |
| At ages 16 or less (ref.)  At ages 17+ | 1.32 | .346 | 0.41 | .141 | 0.96 | .892 | 1.02 | .867 | 0.99 | .950 | 1.25 | .360 |
| **Family structure** | Joint test *p =* .130 | | | | | | Joint test *p =* .632 | | | | | |
| Living with both parents (ref.)  Not living with both parents | 1.67 | .125 | 0.86 | .787 | 0.74 | .436 | 0.88 | .329 | 0.99 | .983 | 0.78 | .364 |
| **Housing tenure** | Joint test *p =* .926 | | | | | | Joint test *p =* .079 | | | | | |
| Owning (ref.)  Not owning | 0.84 | .784 | 0.78 | .677 | 0.77 | .525 | 0.83 | .150 | 0.97 | .868 | **0.47** | **.019** |
|  |  |  |  |  |  |  |  |  |  |  |  |  |

Estimates are relative risk ratios (RRR) from bivariate multinomial logistic models adjusted for complex sampling and non-response. Reference category is having never initiated e-cigarettes. Bolded estimates are statistically significant at the .05 level. “Joint test *p*” refer to the joint test of significance of coefficients across the 3 outcome categories using the Stata *test* command.

**SUPPLEMENTARY TABLE 2**

**Socioeconomic distribution of smoking status. Next Steps study, 2015-16.**

|  |  |  |  |  |
| --- | --- | --- | --- | --- |
|  | **Never**  **smokers**  *n* = 4,306 | **Former**  **smokers**  *n* = 1,202 | **Non-daily**  **smokers**  *n* = 818 | **Daily**  **smokers**  *n* = 1,095 |
|  |  |  |  |  |
| **Variables** | **Weighted %** | **Weighted %** | **Weighted %** | **Weighted %** |
|  |  |  |  |  |
| **Education (NVQ)**  No qualifications  1-2 – Secondary  3 – A-levels  4-5 – FE/HE | 34.1  38.9  56.0  63.9 | 19.6  20.2  16.3  16.3 | 11.1  12.1  13.6  10.9 | 35.1  28.7  14.2  8.9 |
| **Social class (NS-SEC)**  1-2 - Higher  3-4 - Intermediate  5-7 - Lower  Not applicable | 58.8  53.4  43.0  41.5 | 16.5  19.6  19.0  18.5 | 12.8  11.0  12.8  9.8 | 11.9  16.0  25.2  30.2 |
| **Employment status**  FT employed  PT employed  Unemployed  Other | 53.5  44.1  39.8  42.9 | 17.5  21.8  14.1  20.7 | 12.7  10.9  10.3  9.4 | 16.3  23.3  35.7  27.1 |
|  |  |  |  |  |

NVQ: National Vocational Qualification. FE/HE: Further or higher education. NS-SEC: National Statistics Socio-economic Classification. FT: Full-time. PT: Part-time.

**SUPPLEMENTARY TABLE 3**

**Distribution of e-cigarette use and cigarette smoking. Next Steps (*n* = 7,419, 2015-16).**

|  | **E-cigarette use** | | | |
| --- | --- | --- | --- | --- |
| **Smoking status** | Never user | Former user | Non-daily user | Daily user |
|  |  |  |  |  |
| Frequency | N | N | N | N |
| Never smoker | 4,078 | 207 | 14 | 5 |
| Former smoker | 687 | 345 | 52 | 118 |
| Non-daily smoker | 340 | 324 | 85 | 69 |
| Daily smoker | 318 | 605 | 140 | 32 |
| Weighted prevalence | Row % | Row % | Row % | Row % |
| Never smoker | 94.8 | 4.7 | 0.3 | 0.2 |
| Former smoker | 54.1 | 29.6 | 4.6 | 11.6 |
| Non-daily smoker | 39.3 | 40.3 | 11.2 | 9.1 |
| Daily smoker | 29.8 | 54.3 | 13.0 | 2.8 |
